# Interfacial Elemental Analysis of Slanted Edge-Contacted Monolayer MoS_2_ Transistors via Directionally Angled Etching

**DOI:** 10.1021/acsnano.4c13581

**Published:** 2025-01-21

**Authors:** Chia-Chun Lin, Naomi Tabudlong Paylaga, Chun-Chieh Yen, Yu-Hsuan Lin, Kuang-Hsu Wang, Kenji Watanabe, Takashi Taniguchi, Chi-Te Liang, Shao-Yu Chen, Wei-Hua Wang

**Affiliations:** 1 Molecular Science Technology Program, Taiwan International Graduate Program, Academia Sinica, Taipei 115201, Taiwan; 2 National Central University, Zhongli, Taoyuan 320317, Taiwan; 3 Institute of Atomic and Molecular Sciences, Academia Sinica, Taipei 106319, Taiwan; 4 Department of Physics, National Taiwan University, Taipei 106319, Taiwan; 5 Research Center for Electronic and Optical Materials, National Institute for Materials Science, 1-1 Namiki, Tsukuba 305-0044, Japan; 6 Research Center for Materials Nanoarchitectonics, National Institute for Materials Science, 1-1 Namiki, Tsukuba 305-0044, Japan; 7 Taiwan Semiconductor Research Institute (TSRI), Hsinchu 300091, Taiwan; 8 Center of Atomic Initiative for New Materials, National Taiwan University, Taipei 106319, Taiwan; 9 Center for Condensed Matter Sciences, National Taiwan University, Taipei 106319, Taiwan

**Keywords:** directional etching, edge contact, transition metal dichalcogenides, elemental analysis, interfacial chemical property

## Abstract

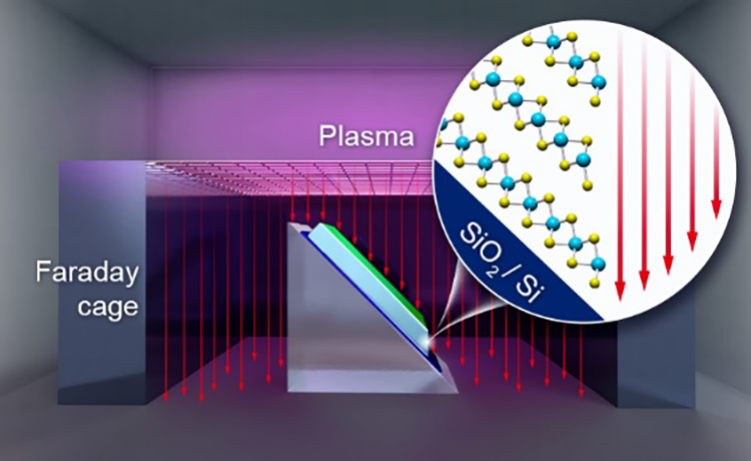

Edge contacts offer a significant advantage for enhancing the performance of semiconducting transition metal dichalcogenide (TMDC) devices by interfacing with the metallic contacts on the lateral side, which allows the encapsulation of all of the channel material. However, despite intense research, the fabrication of feasible electrical edge contacts to TMDCs to improve device performance remains a great challenge, as interfacial chemical characterization via conventional methods is lacking. A major bottleneck in explicitly understanding the chemical and electronic properties of the edge contact at the metal–two-dimensional (2D) semiconductor interface is the small cross section when characterizing nominally one-dimensional edge contacts. Here, we demonstrate a directional angled etching technique that enables the characterization of the interfacial chemistry at the metal–MoS_2_ junction when in an edge-contact configuration. The slanted edge structure provides a substantial cross section for elemental analysis of the edge contact by conventional X-ray photoemission spectroscopy, in which a simple chemical environment and sharp interface were revealed. Facilitated by the well-characterized contact interface, we realized slanted edge-contacted monolayer MoS_2_ transistors encapsulated by hexagonal boron nitride. The transport characteristics and photoluminescence of these transistors allowed us to attribute the efficient carrier injection to direct and Fowler–Nordheim tunneling, validating the distinct Au–MoS_2_ interface. The established method represents a viable approach to fabricating edge contacts with encapsulated 2D material devices, which is crucial for both the fundamental study of 2D materials and high-performance electronic applications.

## Introduction

Electrical contact is the key factor for assessing the electronic properties of two-dimensional (2D) materials.^1,2^ Efficient electrical contact with 2D semiconductors (2DSs) encapsulated by van der Waals (vdW) heterostructures is important for both fundamental research and practical applications.^3−5^ The top contact approach is straightforward for 2D geometry and has been widely developed. On the other hand, edge contact with 2DSs has several advantages over top contact.^3,4,6−8^ First, despite significant advances in channel length reduction, contact length scaling remains an issue for transistor scaling owing to the large transfer length in the top contact. Conversely, edge contact is unaffected by contact scaling.^9^ Second, 2DSs exhibit a highly anisotropic crystal structure, with the edge sites and basal planes having very different chemical and structural properties. In particular, methods that have been extensively developed for conventional semiconductors can be employed in edge contact to control chemical bonding at the metal–semiconductor interface.^4,10−12^ Third, for practical purposes, edge contact naturally endows devices with desirable configurations, such as encapsulated vdW heterostructures for protecting chemically sensitive 2D materials,^4,13^ efficient carrier modulation,^14,15^ and uniform carrier transport.^6^

**Figure 1:**
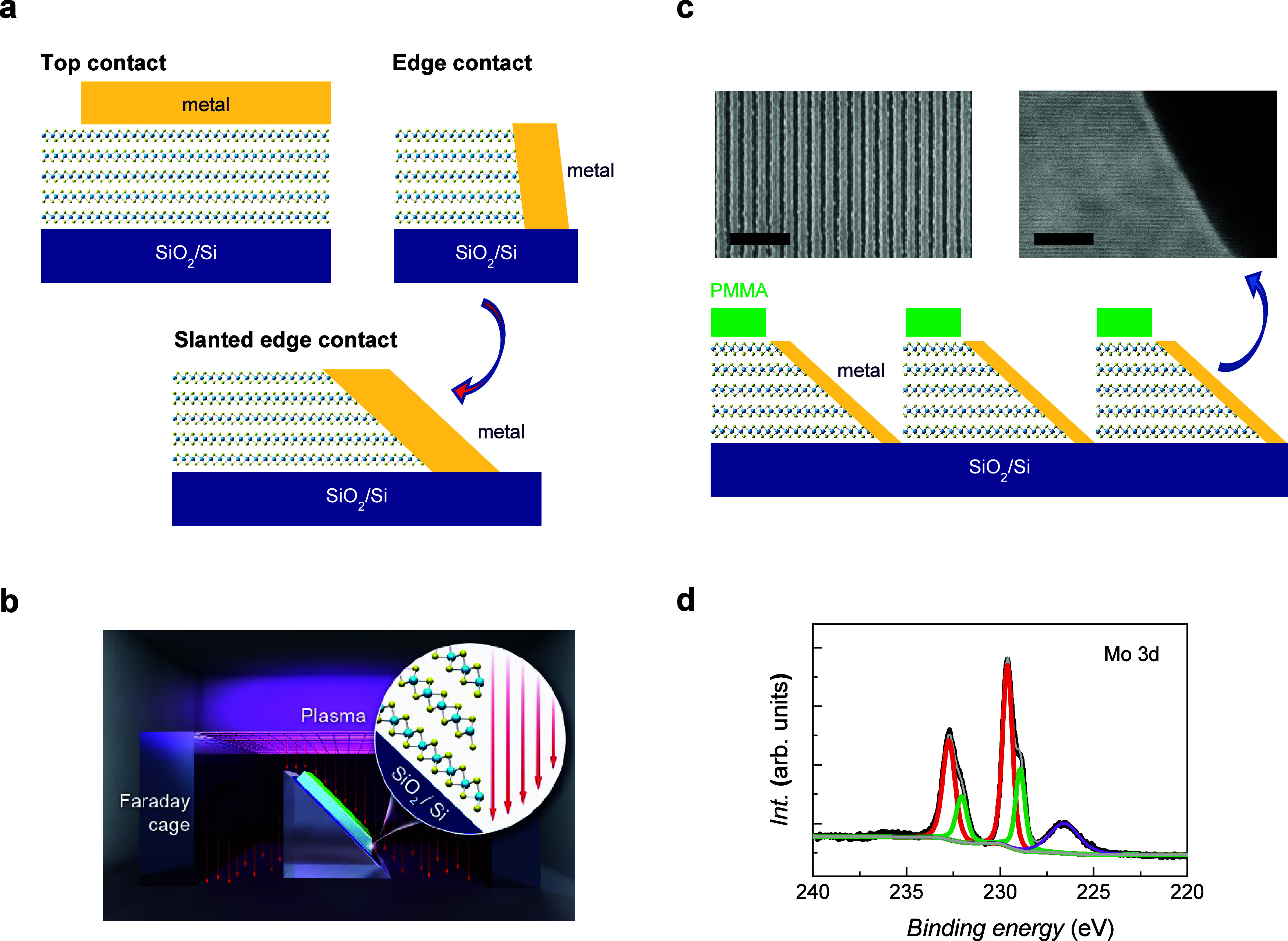
(a) Schematic of the comparison of the top, edge, and SE contacts. (b) Schematic of directionally angled etching to fabricate a MoS_2_ SE sample. The trajectory of the incident plasma ions is preferentially collimated by the Faraday cage. (c) Schematic of the periodic SE of MoS_2_ for XPS analysis. Left inset: TEM image of the cross section of the SE, which shows an extended, uniform edge for chemical analysis via XPS. The scale bar is 5 μm. Right inset: SEM image of the periodic SE structure of the bulk MoS_2_ sample. The scale bar is 10 nm. (d) Typical XPS spectrum of a MoS_2_ sample fabricated with a periodic SE structure covered by a Au layer.

Examining the interfacial chemistry at the 2DS contact is essential for understanding the contact properties, including the contact resistance and Fermi level pinning, and further improving the reliability of edge-contact devices.^2^ Previously, studies on the chemical bonding that occurs at the metal–2DS interface have focused on top contacts because of the direct characterization of the surface chemistry at the 2D interface.^16−18^ For edge contact via plasma etching, several issues can occur, including the generation of defects or dangling bonds, chemical termination by oxygen,^19^ and the formation of chemical bonds at the metal–2DS edge interface, which critically affect the electrical performance of edge-contacted devices and hinder optimization of the edge contact properties.^6^ However, investigating the metal–2DS interface in edge-contact geometry is very challenging because of the very small cross section when characterizing nominally one-dimensional metal contacts, as depicted in the upper left panel of Figure 1a, which hampers the advancement of the edge contact approach. Here, we present a directional angled etching technique to fabricate a slanted edge (SE) structure on MoS_2_ to explicitly reveal the chemical information on the metal–MoS_2_ edge interface in the edge-contact configuration. Specifically, we designed a homemade Faraday cage that encloses the samples in a reactive ion etching (RIE) system for directional etching with an SF_6_/Ar plasma. As depicted in the lower panel of Figure 1a, the resulting SE structure yields a substantial cross section for elemental analysis of the SE contact by conventional X-ray photoemission spectroscopy (XPS),^20,21^ which revealed a sharp metal–MoS_2_ interface. Moreover, after characterizing the interface between the metal and MoS_2_ edge, we successfully demonstrated a high-quality SE contact in the edge-contacted monolayer (ML) MoS_2_ transistors encapsulated by hexagonal boron nitride (h-BN).

## Results and Discussion

Transition metal dichalcogenides (TMDCs) are an important class of 2DSs that show great promise for electronics and photonics applications.^22−24^ To yield a controllable SE structure for surface characterization and subsequent electrical contact with TMDCs, we developed a MoS_2_ directional etching method by employing a Faraday cage,^25,26^ as depicted in Figure 1b (Supporting Information S1). The trajectory of the incident plasma ions is preferentially collimated by the geometric design of the Faraday cage, yielding various material systems with slanted angle profiles under plasma processing conditions.^27−30^ The advantages of using this technique are 2-fold. First, the etching angle is highly controllable and can be arbitrarily adjusted.^27,31^ For example, for surface characterization by XPS, we choose an angle of approximately 45° to optimize the cross section of the XPS signal. Second, the use of a Faraday cage is beneficial as the collimated ions can create a uniform edge during the etching process. A schematic of the periodic SE of MoS_2_ for XPS analysis is depicted in Figure 1c. We employed e-beam lithography to fabricate a large array of closely spaced SEs to increase the size of the cross section for subsequent XPS surface characterization, as shown in the scanning electron microscopy (SEM) image (left inset of Figure 1c). When used as an etching mask during the etching process, the periodic array of poly(methyl methacrylate) (PMMA) strips fully suppresses the planar MoS_2_ XPS signal, indicating that all of the observed XPS signals are due to the SEs. The right inset of Figure 1c shows the transmission electron microscopy (TEM) image of the SE, which is extended and uniform for an accurate chemical analysis. Figure 1d shows a typical XPS spectrum of a MoS_2_ sample with a periodic SE structure. Notably, a pronounced XPS signal was attained by employing conventional XPS, which facilitates the characterization of the interfacial chemistry and optimization of the SE contact. In addition to XPS, our method can be utilized to identify edge characteristics via other surface characterization tools, such as scanning tunneling microscopy, Raman spectroscopy, and Fourier transform infrared spectroscopy.

**Figure 2:**
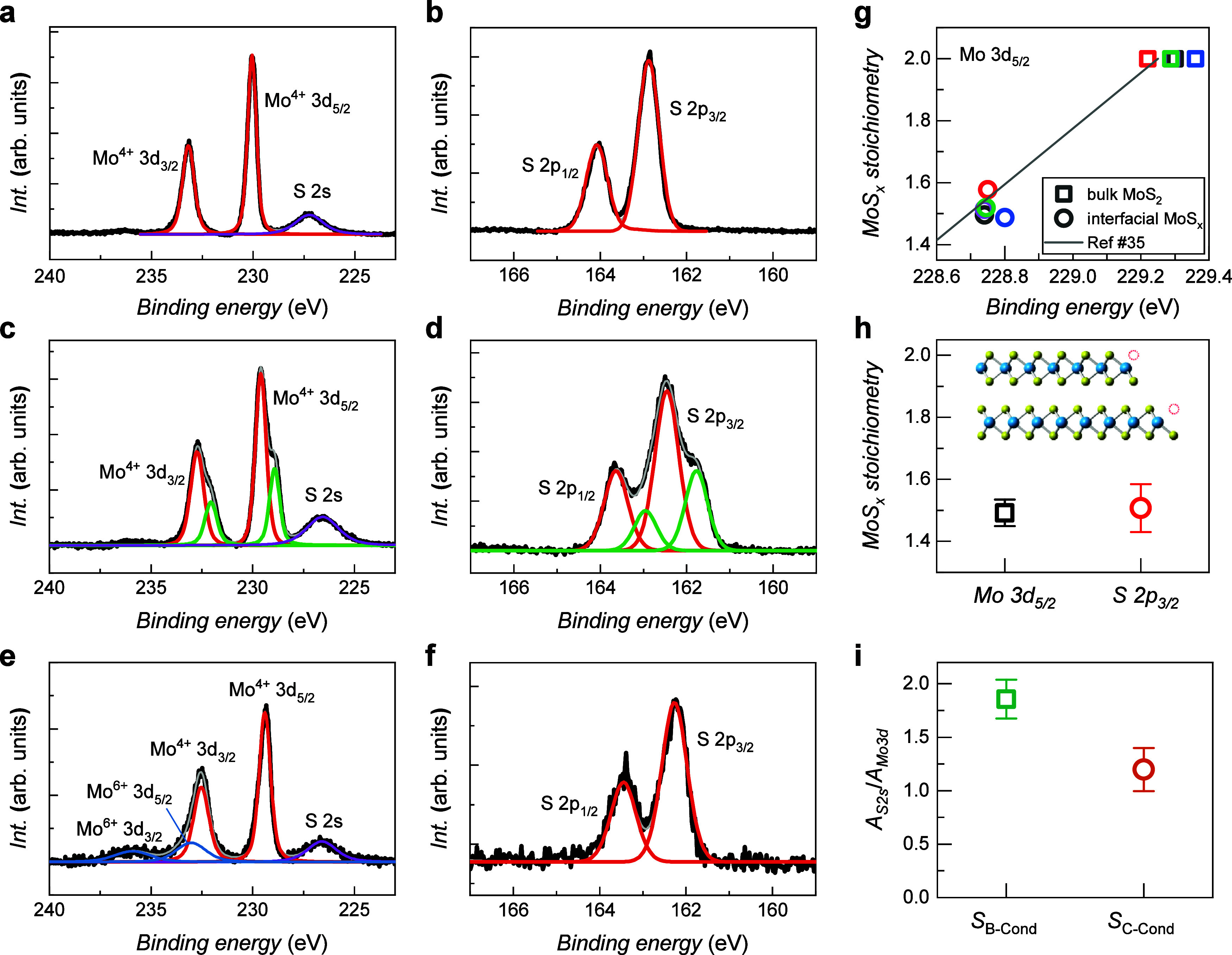
XPS Mo 3d and S 2p spectra of (a, b) pristine MoS_2_, (c, d) MoS_2_ SE sample prepared via the optimized etching process, and (e, f) MoS_2_ SE sample exposed to ambient conditions for 20 min after etching. (g) Stoichiometry of the MoS_2_ SE surface as a function of the binding energy of the Mo 3d_5/2_ peak. (h) Estimation of the stoichiometry of the MoS_2_ SE surface calculated from the shifts in the binding energies of the Mo 3d_5/2_ and S 2p_3/2_ peaks. The error bar was determined from spectra acquired from samples processed under the same conditions. (i) Comparison of the ratios of the peak areas of the core levels corresponding to S 2s to Mo 3d between samples fabricated via minimized oxidation (*S*_B–Cond_) and samples prepared via prolonged oxidation (*S*_C–Cond_).

To exemplify our method, we show that by employing conventional XPS, we can uncover the interfacial chemistry at the SE contact of MoS_2_, which is deterministically affected by the etching of the edge contact. We compare the Mo 3d and S 2p core levels for three MoS_2_ samples, designated A, B, and C, respectively: pristine bulk MoS_2_, MoS_2_ SE etched by SF_6_, and MoS_2_ SE etched by SF_6_ with prolonged oxidation under ambient conditions for 20 min. Au (3 nm) was deposited on all the MoS_2_ SE edge samples to encapsulate the surface and protect against natural oxidation^16^ while still allowing the collection of XPS signals. The area of the periodic SE structure is larger than the spot size of the X-ray incident beam (approximately 100 μm in diameter) to maximize the XPS signal. The Shirley inelastic background was subtracted from all XPS data^32^ for clear presentation even though the XPS signals are dominant. As a reference, we show the XPS spectra of a controlled MoS_2_ sample with a pristine surface (sample A) in Figure 2a,b. The XPS spectra of pristine MoS_2_ exhibit doublets due to the Mo 3d and S 2p peaks at 230.0 (Mo 3d_5/2_) and 162.8 eV (S 2p_3/2_), respectively. These binding energies (BEs) are consistent with pristine MoS_2_ peaks reported previously.^33^ All of the Mo 3d and S 2p doublets were analyzed with compliance to the constraints of area, full width at half-maximum (fwhm), and position considering spin–orbit coupling.^34^

Next, we discuss the XPS spectra of the MoS_2_ SE samples etched by SF_6_, as shown in Figure 2c,d. The Mo 3d_5/2_ doublet at 229.6 eV (red) can be attributed predominantly to bulk MoS_2_ with minor doping.^35^ Remarkably, a well-resolved doublet (green) with a BE of ∼0.7 eV lower than that of the doublet corresponding to the underlying bulk MoS_2_ is clearly observed. These XPS spectra are invariant across the SE samples, indicating uniform chemical conditions at the interface after SE etching. The decrease in BE is in line with p-type doping in the substoichiometric MoS_*x*_ layer^35,36^ and suggests the presence of undercoordinated Mo upon removal of negatively charged S^2–^ ions during the etching process. For the MoS_2_ basal plane, S vacancies originate from preexisting structural defects^17^ and can be caused by various treatments,^33,37^ as evidenced by the decrease in the BE determined by XPS. Moreover, MoS_2_ with S vacancies created by ion bombardment shows p-type doping, as exemplified by scanning tunneling spectroscopy.^38^ For the S peak, a well-resolved S 2p_3/2_ doublet at 161.8 eV is observed, which corresponds to a BE of ∼1.0 eV lower than that of bulk MoS_2_, as shown in Figure 2d. This additional S 2p doublet can be ascribed to the monosulfide,^39^ supporting the presence of S vacancies at the MoS_2_ SE edge interface. One may consider that the decrease in the BE is associated with the formation of metallic Mo. However, we did not observe a larger decrease in the BE of ∼1.1 eV corresponding to the reduction of Mo^4+^ to Mo^0^,^16,18^ excluding the possibility that the decrease in the BE is associated with the formation of metallic Mo.

The surface structure of the MoS_2_ SE can be determined by further analysis of the XPS spectra. The probing depth of XPS measurements is typically 6–9 nm,^40^ and the capping Au layer is 3 nm thick. Considering the ratio of the intensity of the bulk MoS_2_ layer to that of the surface layer, it can be estimated that the substoichiometric MoS_*x*_ layer is very thin, at approximately 1 nm. Notably, the bulk MoS_2_ crystal structure extends well to the edge and is unaffected during etching. The basal plane of MoS_2_ requires well-controlled ion sputtering at a grazing angle to etch the top ML.^37,41^ Importantly, we show that by controlling the ion sputtering alignment in the SE geometry plasma etching at the SE naturally occurs at a grazing angle, leading to a substoichiometric MoS_*x*_ layer that is restrained at the interface. Furthermore, the well-resolved split Mo 3d and S 2p doublets allow us to estimate the stoichiometry of the interfacial MoS_*x*_ layer. We first analyze the Mo BE by using the underlying bulk MoS_2_ as a reference. As S atoms are removed from MoS_2_, the Mo BE decreases, which reflects the changing electrostatic environment of the Mo atoms. By adapting the relationship of the MoS_*x*_ stoichiometry as a result of the S vacancies and Mo BE,^35^ we plot the distribution of our MoS_2_ SE sample by referencing the underlying bulk MoS_2_ (Figure 2g). Here, it is assumed that the shift in the S 2p_3/2_ peak BE exhibits the same energy dependence as the Mo 3d_5/2_ peak because the shift in BE is caused by Fermi level alteration. Figure 2h shows the stoichiometry of the MoS_*x*_ layer in all of the SE samples, which were estimated by the shifts in BEs of both the Mo 3d_5/2_ peak and the S 2p_3/2_ peak, yielding comparable S vacancies and a MoS_*x*_ stoichiometry of approximately 1.5.

Notably, the fwhm of the Mo 3d_5/2_ doublet is 0.7 eV, which is comparable to the value of 0.6 eV for bulk MoS_2_. Similarly, the fwhm of the additional S 3p_3/2_ doublet is 0.6 eV, which is comparable to that of the S 3p_3/2_ doublet corresponding to the inner MoS_2_. Because the XPS signal reflects the chemical conditions, the peak corresponding to the surface of the MoS_2_ edge can broaden after ion bombardment due to the creation of disorders.^33,35^ The absence of peak broadening in this work suggests that the substoichiometric MoS_*x*_ layer has a uniform chemical environment that is comparable to that of crystalline MoS_2_. This insignificant broadening may be attributed to negligible disorders or metallic Mo nanoparticles^42^ in the interfacial MoS_*x*_ layer. Generally, the MoS_2_ edge produced by plasma etching can have many dangling bonds and extensive disorder, which subsequently lead to the formation of Mo oxides in the ambient environment. To assess the extent of MoS_2_ SE oxidation, we compared the XPS spectra of sample C, which was kept under ambient conditions (20 °C and 50% relative humidity) for 20 min after SE etching and before being transferred to the evaporation chamber, with those of sample B, where the exposure time was limited to approximately 1 min. Figure 2e,f shows the Mo 3d and S 2p spectra of sample C, respectively, revealing dominant Mo 3d and S 2p doublets. The Mo 3d_5/2_ doublet at 229.4 eV indicates the presence of pristine MoS_2_, which is similar to the SE surface of sample B. Moreover, a small Mo 3d_5/2_ doublet at 233.0 eV arises, indicating the presence of Mo^6+^, which is ascribed to the oxidation of Mo^4+^ (Supporting Information S2).^43,44^ The Mo 3d_5/2_ doublet corresponding to the substoichiometric MoS_*x*_ layer is absent in sample C, suggesting its oxidation upon prolonged exposure. For S, the XPS peaks at higher energies corresponding to S–O bonds and polysulfide species^39,45^ were not observed in either samples B or C. Importantly, this comparison indicates that while the samples are exposed to ambient air, the oxidation of the etched MoS_2_ SE can be negligible if the sample transfer time is short (Supporting Information S3).

To examine the effect of oxidation under ambient conditions on the chemical composition of the MoS_2_ SE, we compared the ratio of the core level peak areas corresponding to S 2s to that of Mo 3d, *A*(S 2s)/*A*(Mo 3d), between *S*_B–Cond_ and *S*_C–Cond_, which are plotted in Figure 2i. *S*_B–Cond_ and *S*_C–Cond_ correspond to the samples fabricated via the same oxidation conditions as those used for samples B and C, respectively. For the area of the Mo 3d peak, all of the doublets originating from bulk MoS_2_, the substoichiometric MoS_*x*_ layer, and the MoO_3_ interfacial layers were included. The ratio *A*(S 2s)/*A*(Mo 3d) in the *S*_C–Cond_ samples is significantly lower than that in the *S*_B–Cond_ samples, suggesting that the S atoms in the MoS_*x*_ SE layer are replaced by the O atoms. Notably, sample B does not exhibit any features other than the XPS peaks corresponding to bulk MoS_2_ and the substoichiometric MoS_*x*_ layer, indicating the absence of detectable interfacial chemical reactions (Supporting Information S2). Generally, for sample B and even for sample C, the extent of oxidation at the SE of the MoS_2_ layer is low. Moreover, the XPS spectra of the MoS_2_ SE sample provide both a quantitative benchmark to assess the quality of the MoS_2_ SE contact discussed later and direct feedback to optimize the SE contact.

**Figure 3:**
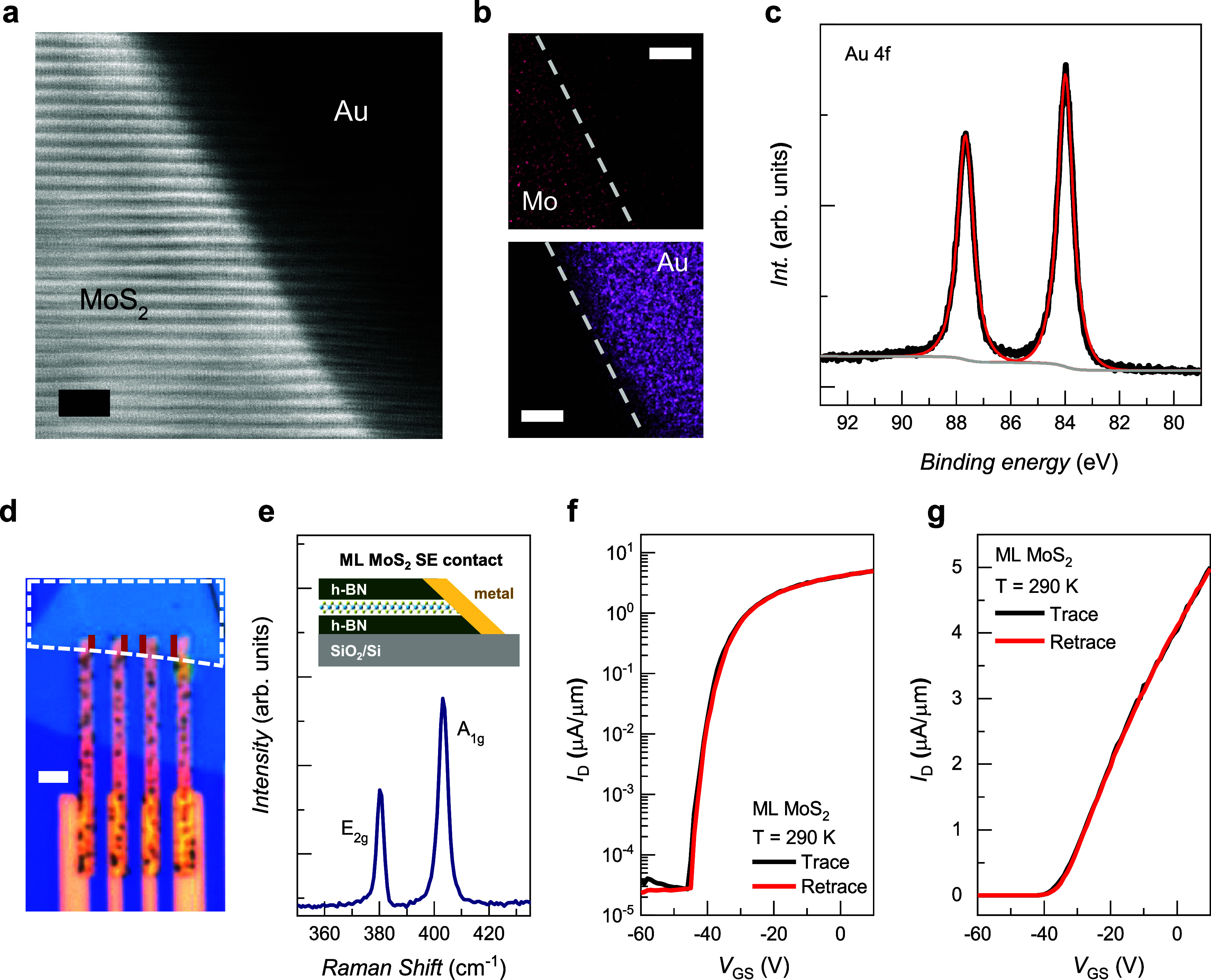
(a) Cross-sectional STEM image of a MoS_2_ SE sample covered with 100 nm of Au. The crystalline MoS_2_ layers are preserved up to the Au–MoS_2_ interface. The scale bar is 2 nm. (b) Energy-dispersive X-ray spectroscopy image of the spatial distribution of Mo and Au in the MoS_2_ SE sample. The scale bar is 5 nm. (c) Au 4f XPS spectrum indicating that no bonding occurred between MoS_2_ and Au. (d) Typical optical micrograph of a slanted edge-contacted ML MoS_2_ field-effect transistor. The red line indicates the SE, and the white dashed line marks the area of ML MoS_2_. The scale bar is 2 μm. (e) Raman spectrum of the ML MoS_2_ sample. Transfer curves for the SE-contacted ML MoS_2_ transistor (sample D) with *I*_*D*_ on the (f) logarithmic and (g) linear scales at *T* = 290 K.

The interfacial chemistry of the metal–MoS_2_ SE interface can be further examined by considering the chemical state of the Au layer at the interface. Figure 3a shows a TEM image of the cross section of the SE, revealing a clear interface between the crystalline MoS_2_ SE and the Au layer. Remarkably, the crystalline structure of MoS_2_ is well preserved up to the Au–MoS_2_ SE interface, which substantiates the inference in the XPS analysis in Figure 2. The darker crystalline structure (to the left of the interface) is ascribed to the SE not being perpendicular to the imaging plane. The energy-dispersive X-ray spectroscopy image confirms the clear separation of the MoS_2_ SE and the Au layer in the interface region, as shown in Figure 3b. The disorder and metal penetration commonly observed after deposition are absent, corroborating that the highly crystalline state extends into the Au–MoS_2_ SE interface at an atomic resolution. To probe the interfacial chemistry of the Au–MoS_2_ SE interface, we present the XPS spectrum of the Au 4f core level of sample B, as shown in Figure 3c. Pronounced Au 4f_7/2_ doublet at 84.0 eV is observed, indicating the dominance of metallic gold. XPS peaks corresponding to other chemical states of Au, e.g., Au–S bonding^46^ and Au nanoparticles,^39^ are absent, indicating that the Au layer interfaced with the MoS_2_ SE without the formation of chemical bonds.

To understand more than simply the chemical information about the Au–MoS_2_ SE interface, we acquire correlated electrical characteristics by fabricating a SE-contacted ML MoS_2_ transistor. Details regarding the fabrication of the SE-contacted ML MoS_2_ transistors are given in Supporting Information S4. Figure 3d shows an optical micrograph of a typical SE-contacted ML MoS_2_ transistor. The SE is fabricated under the same etching conditions as those used for sample B for the XPS measurements. In contrast to the bulk MoS_2_ from the XPS analysis, the SE in the device is dominated by h-BN encapsulating ML MoS_2_, as depicted in the inset of Figure 3e (Supporting Information S5). Figure 3e shows the Raman spectrum of the MoS_2_ device, revealing peaks at 380.4 and 403.4 cm^–1^, which correspond to the *E*_2*g*_^1^ and *A*_1*g*_ vibrational modes, respectively. The two Raman peaks are separated by 20 cm^–1^, indicating that the MoS_2_ sample is ML,^47^ which is also validated by the photoluminescence (PL) spectra of the MoS_2_ device shown in Figure 5. We now discuss the transport characteristics of the SE-contacted ML MoS_2_ transistor. Figure 3f shows the logarithmic transfer curve, log(*I*_*D*_), as a function of *V*_*GS*_ for the SE-contacted ML MoS_2_ transistor (sample D) at *T* = 290 K. Current annealing is conducted on the MoS_2_ SE samples to improve the electrical characteristics (Supporting Information Figure S6). Sample D manifests an ideal turn-on characteristic with a large on/off ratio of 1 × 10^5^ and a subthreshold swing of 1 V/dec Figure 3g shows *I*_*D*_ as a function of *V*_*GS*_ for sample D on a linear scale at *T* = 290 K. The threshold is approximately −35 V, indicating n-type transport behavior, which is in line with the n-type MoS_2_ commonly observed.^3,48^ Notably, the hysteresis of the transfer curve is negligible, indicating the absence of trap states near the Fermi level in both the Au–MoS_2_ SE contact and the ML MoS_2_ channel.^49−51^ The absence of trap states substantiates the presence of a clean Au–MoS_2_ SE interface, which is also evidenced by XPS and TEM analysis. Moreover, these findings confirm the effectiveness of h-BN encapsulation to protect against contamination from ambient molecules. Markedly, the SE-contacted ML MoS_2_ transistor exhibits a high current density of 5 μA/μm at *V*_*GS*_ = 10 V, indicating an effective electrical contact.

**Figure 4:**
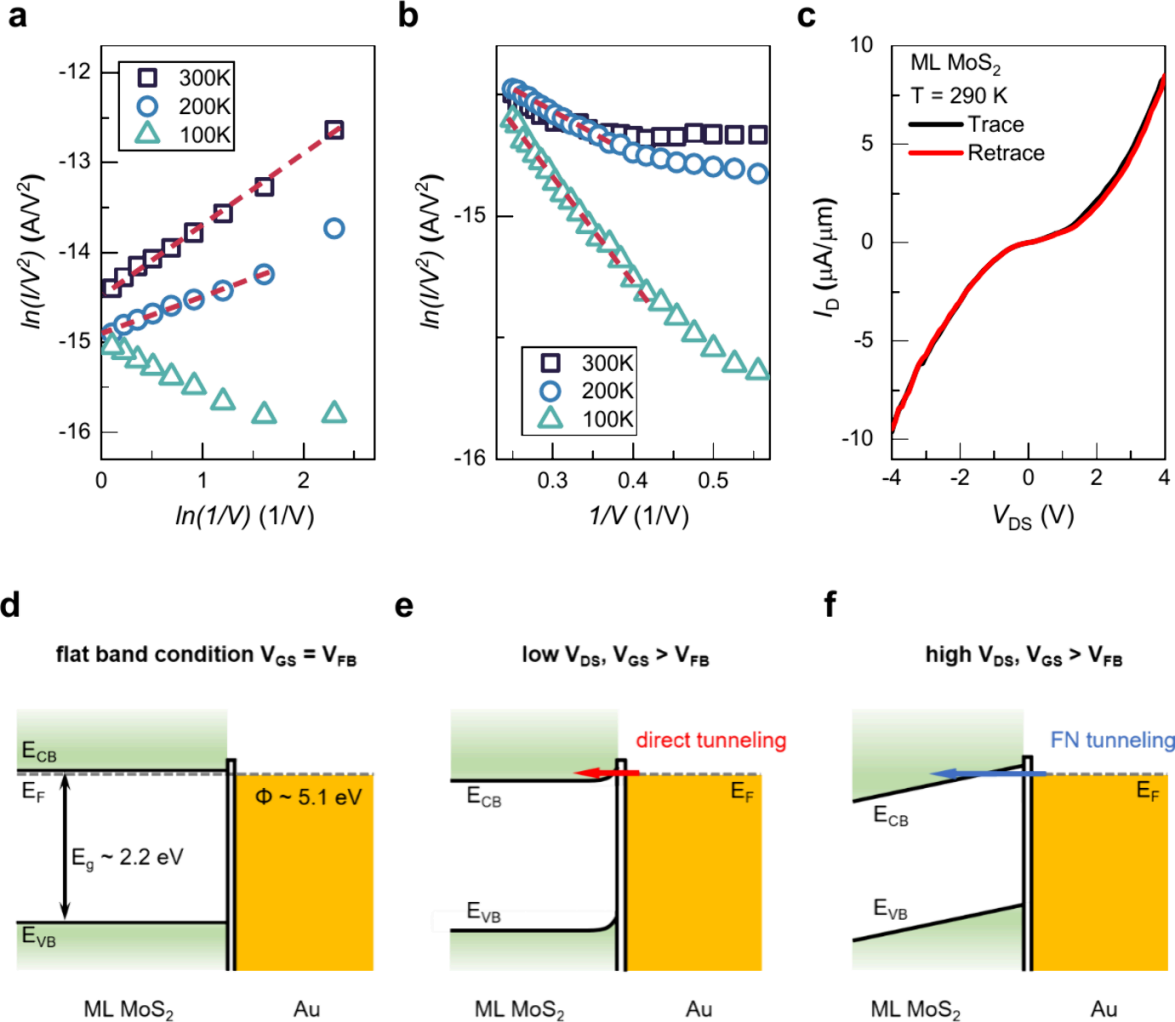
Current–voltage scaling relationships of (a)  versus ln(1/*V*_*DS*_) and (b)  versus −1/*V*_*DS*_ for sample D at different temperatures and *V*_*GS*_ = 60 V. The clear linear current–voltage scaling relationship indicates that, at low *V*_*DS*_, direct tunneling governs charge transport across the Au–ML MoS_2_ SE contact. At high *V*_*DS*_, the charge injection mechanism can be attributed to Fowler–Nordheim emission. (c) Output curve for sample D with *V*_*GS*_ = 60 V at *T* = 290 K. Schematics of the band diagram of the charge injection mechanism (d) for the flat band condition (*V*_*GS*_ = *V*_*FB*_), (e) at low *V*_*DS*_, and (f) at high *V*_*DS*_. At low *V*_*DS*_, direct tunneling with negligible thermionic emission indicates a very small energy barrier. At high *V*_*DS*_ and when *V*_*GS*_ > *V*_*FB*_, FN tunneling dominates charge injection owing to thinning of the barrier.

**Figure 5:**
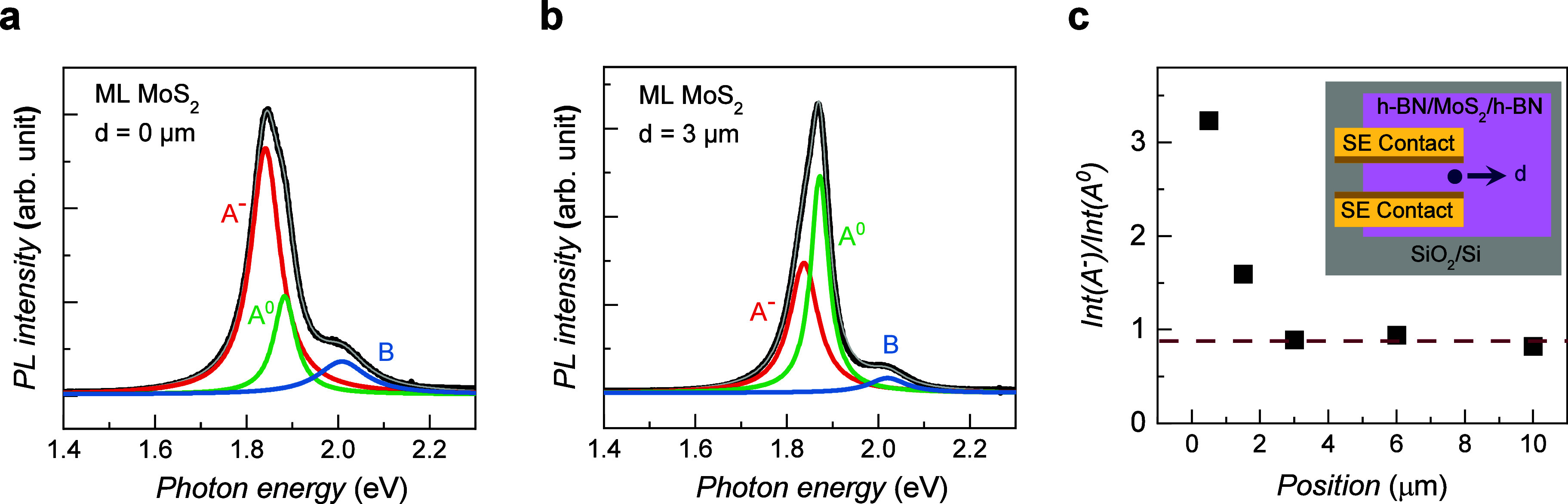
Position-dependent PL spectra of the ML MoS_2_ SE device measured (a) at the SE contact (*d* = 0 μm) and (b) away from the SE contact (*d* = 3 μm). (c) Ratio of the integrated intensities of the trion to the A exciton as a function of position from the SE contact. The ratio remains at 0.9 away from the SE contact and starts to increase when it approaches the SE contact, reaching 3.2 near the SE contact.

We now discuss the metal–ML MoS_2_ SE contact by examining the electrical conduction mechanism. To understand the charge injection and transport mechanism across the Au–ML MoS_2_ SE contact, we consider direct tunneling^52^ at a low *V*_*DS*_ and Fowler–Nordheim (FN) tunneling^53^ at a high *V*_*DS*_, where the current–voltage scaling relationships are described by  and , respectively. Figure 4a shows the plot of  versus ln(1/*V*_*DS*_) for sample D at different temperatures with *V*_*GS*_ = 60 V. At *T* = 300 and 200 K, the current–voltage relationship is clearly linear, which indicates that direct tunneling governs charge transport across the Au–ML MoS_2_ SE contact. The band alignment at the Au–ML MoS_2_ SE contact is depicted in Figure 4d. The band gap of ML MoS_2_ is estimated to be *E*_*g*_ = 2.2 eV.^54,55^ The ML MoS_2_ channel is strongly n-type, as evidenced by the transfer characteristics and PL measurements (Figure 5). The direct tunneling is observed over the entire *V*_*DS*_ range at *T* = 300 K, reaching a value as small as 0.1 V. The direct tunneling observed at *T* ≥ 200 K indicates a very small energy barrier corresponding to thermionic emission (Supporting Information S7), as depicted in Figure 4e, facilitating a tunneling current at the contact interface at low *V*_*DS*_. In addition, the transport behavior at *T* = 100 K can no longer be described by the positive slope, because the direct tunneling is completely suppressed when thermionic emission decreases.

Figure 4b presents the plot of  versus −1/*V*_*DS*_ for sample D with *V*_*GS*_ = 60 V at different temperatures. At high *V*_*DS*_, the charge injection mechanism can be attributed to FN emission, as evident by the characteristic negative slopes in the current–voltage relationship at different temperatures.^56^ The dominance of FN emission over the thermionic emission contribution can be ascribed to large band bending at the interface with a large *V*_*DS*_, as depicted in Figure 4f. Furthermore, the *V*_*DS*_ range of the FN emission increases as the temperature decreases. This is reasonable because as the thermionic emission current decreases, the contribution of the FN emission to the total charge injection increases. Figure 4c shows the output curve, *I*_*D*_ as a function of *V*_*DS*_, for sample D with *V*_*GS*_ = 60 V and *T* = 290 K. The output curve is nonlinear, which is consistent with tunneling behavior.^52,57^ Generally, electrical transport is determined by carrier conduction in the channel and carrier injection across the SE contact. Because electrical transport has been well characterized by the tunneling mechanism, the SE-contacted ML MoS_2_ transistor is governed by injection-limited conduction, suggesting a low channel resistance in the ML MoS_2_ channel encapsulated by h-BN layers. Notably, the electron injection at the edge contact of the ML MoS_2_ transistor is dominated by thermionic/tunneling effects, indicating a thin tunneling barrier for efficient carrier injection (Supporting Information S8).

Finally, we performed PL measurements on the ML MoS_2_ channel region to confirm the doping effect at the contact interface and determine its effect on the electrical properties of the SE contact devices. Figure 5a shows the PL spectrum of ML MoS_2_ adjacent to the SE contact in the ML MoS_2_ transistor (sample D). PL is measured at *d* = 0 μm, which is defined as the distance from the SE contact, as depicted in the inset of Figure 5c. In the PL spectrum, the trion (A^–^), A^0^, and B excitons are identified with PL energies of 1.90, 1.93, and 2.09 eV, respectively.^58^ The strong PL signal and absence of an additional indirect transition clearly indicate ML MoS_2_.^59^ The trion BE is estimated by determining the energy difference between the A^–^ and A^0^ peaks, yielding a value of approximately 35 meV. This trion BE is consistent with that reported for ML MoS_2_,^60^ validating our assignment of sample D as ML MoS_2_. The pronounced PL peak of the trion indicates the presence of an excess of electrons in MoS_2_, which is typical of MoS_2_.^61^Figure 5b shows the PL spectrum of ML MoS_2_ measured at approximately *d* = 3 μm away from the SE contact. Compared with the PL spectra measured at *d* = 0 μm, the ratio of the PL intensity of the A^–^ and A^0^ excitons apparently decreases (Supporting Information S9). To estimate the doping level, we plot the position dependence of the PL intensity ratio of the A^–^ and A^0^ excitons, which is sensitive to the density of nonequilibrium electrons associated with the doping level,^62^ in Figure 5c. The ratio remains at 0.9 °C from the SE contact and starts to increase when it approaches the SE contact, reaching 3.2 °C near the SE contact. This strong n-type doping in the channel of the ML MoS_2_ transistor corroborates the n-type conduction determined from the transport measurement. Moreover, n-type doping near the SE contact may be attributed to efficient charge injection at the SE contact due to the tunneling effect.

## Conclusions

In summary, by fabrication of a periodic SE structure, conventional XPS can be utilized to investigate the metal–MoS_2_ interface at the edge contact. The SE structure is realized by the developed directional etching technique that enables feasible elemental analysis of the edge contact, yielding explicit chemical bonding conditions at the Au–MoS_2_ SE interface. We present distinct XPS spectra from the MoS_2_ SE, which reveals pristine bulk MoS_2_ and an atomically thin substoichiometric MoS_*x*_ surface layer at the Au–MoS_2_ SE interface. Facilitated by the well-characterized Au–MoS_2_ interface, we achieve high-quality contact in the SE-contacted ML MoS_2_ transistors encapsulated by h-BN. By analyzing the temperature-dependent transport characteristics and position-dependent PL of the SE-contacted ML MoS_2_ transistors, we discuss the efficient carrier injection governed by direct and FN tunneling. This edge contact method offers a viable approach to fabricating 2D material devices encapsulated in vdW heterostructures to advance the study of 2D materials for high-performance electronic and multifunctional applications.

## Methods

### Fabrication of the Periodic SE Structure of MoS_2_ for XPS Analysis

We employed e-beam lithography to fabricate a large array of closely spaced SEs to increase the size of the cross section for subsequent XPS surface characterization. The plasma power of the reactive ion etching (RIE) system used for etching is 10 W. The TEM images of the MoS_2_ sample with a periodic SE confirm the extended and uniform SE structure for accurate chemical analysis. When used as an etching mask during the etching process, the periodic array of poly(methyl methacrylate) (PMMA) strips fully suppresses the planar MoS_2_ XPS signal, indicating that all the observed XPS signals are due to the SEs.

### Faraday Cage for Directional Etching of MoS_2_ SE

The dimensions of the Faraday cage and relative sample position are depicted in the Supporting Information. The distance between the sample and the mesh is approximately 2 mm. The Faraday cage including the grid is made of stainless steel. The side length of the square hole is 0.56 mm and the diameter of the grid is 0.2 mm. The general rule to shield the radiation is that the diameter of the grid hole should be less than one-tenth of the wavelength of the radiation. For our RIE system, the wavelength is approximately 22 m corresponding to a radio frequency of 13.56 MHz. The diameter of the grid hole of 0.56 mm is significantly smaller than the wavelength, therefore leading to complete shielding of the RF electric field by the Faraday cage.

## Supporting Information Available

Faraday cage for directional etching of MoS_2_ slanted edge (SE); S 2p spectra of MoS_2_ SE samples after natural oxidation for different durations; XPS analysis of the controlled MoS_2_ SE sample with Cr/Au capping layer; fabrication of the SE-contacted MoS_2_ transistors; slanted edge of h-BN characterized by SEM; Hybrid current/thermal annealing of the SE-contacted MoS_2_ transistors; electrical transport mechanism; using Simmon’s model for tunneling current; power dependence of the PL on the ML MoS_2_ adjacent to the SE contact of the ML MoS_2_ transistor (PDF)
