# Supplementary material for: Interfacial Elemental Analysis of Slanted Edge-Contacted Monolayer MoS2 Transistors via Directionally Angled Etching
Source: ACS Nano. 2025 Jan 21;19(4):4452–61. doi: 10.1021/acsnano.4c13581 (PMC11803913; doi:10.1021/acsnano.4c13581)
Supplement: Supplementary file 1 — nn4c13581_si_001.pdf [file nn4c13581_si_001.pdf]

Supporting Information

Interfacial elemental analysis of slanted edge-contacted monolayer MoS_2 transistors via directionally angled etching

*Chia-Chun Lin[1,2,3], Naomi Tabudlong Paylaga[1,2,3], Chun-Chieh Yen[3†], Yu-Hsuan Lin[4], Kuang-Hsu Wang[4], Kenji Watanabe[5], Takashi Taniguchi[6], Chi-Te Liang[4,7], Shao-Yu Chen[8,9], Wei-Hua Wang[3]**

[1]Molecular Science Technology Program, Taiwan International Graduate Program, Academia Sinica, Taipei 115201, Taiwan

[2] National Central University, Zhongli, Taoyuan 320317, Taiwan

[3] Institute of Atomic and Molecular Sciences, Academia Sinica, Taipei 106319, Taiwan

[4]Department of Physics, National Taiwan University, Taipei 106319, Taiwan

[5]Research Center for Electronic and Optical Materials, National Institute for Materials Science, 1-1 Namiki, Tsukuba 305-0044, Japan

[6]Research Center for Materials Nanoarchitectonics, National Institute for Materials Science, 1-1 Namiki, Tsukuba 305-0044, Japan

[7]Taiwan Semiconductor Research Institute (TSRI), Hsinchu 300091, Taiwan

[8]Center of Atomic Initiative for New Materials, National Taiwan University, Taipei 106319, Taiwan

[9]Center for Condensed Matter Sciences, National Taiwan University, Taipei 106319, Taiwan

S1. The Faraday cage for directional etching of MoS_2 slanted edge (SE)

The dimension of the Faraday cage and relative sample position is depicted in S1a. The distance between the sample and the mesh is approximately 2 mm. The dimension of the grid and the pictures of the angled sample holder and the Faraday cage are shown in Figure S1b-e. The Faraday cage including the grid is made of stainless steel. The side length of the square hole is 0.56 mm and the diameter of the grid is 0.2 mm. The plasma power of the reactive ion etching (RIE) system used for etching is 10 W. The general rule to shield the radiation is that the diameter of the grid hole should be less than one-tenth of the wavelength of the radiation.[1] For our RIE system, the wavelength is approximately 22 m corresponding to a radio frequency of 13.56 MHz. The diameter of the grid hole of 0.56 mm is significantly smaller than the wavelength, therefore leading to complete shielding of the RF electric field by the Faraday cage.

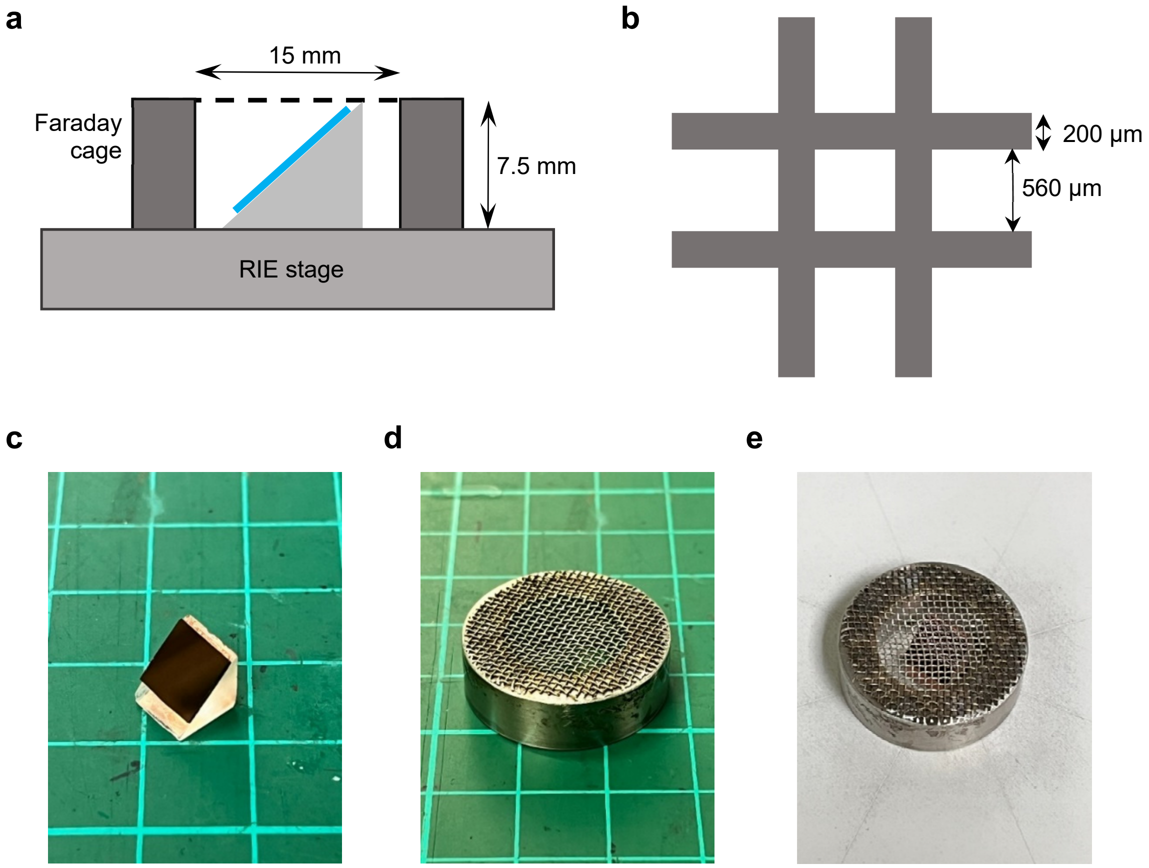

Figure S1. Schematics of (a) Faraday Cage and relative position of the samples, and (b) dimension of the grid. (c) Picture of a sample mounted on a triangular quartz base at a 45° angle. (d, e) Picture of Faraday Cage and sample in the RIE system.

S2. S 2p spectra of MoS_2 SE samples after natural oxidation for different durations

We discuss the S 2p spectra in the main text, as shown in Figure 2b, 2d, and 2f. Here, we discuss the S peaks with higher binding energies (BEs) and present the XPS spectra of samples A, B, and C from the main text in Figure S2a, S2b, and S2c, respectively. For samples A and B, no XPS peaks with higher BEs were observed. In contrast, sample C, after prolonged oxidation under ambient conditions for 20 min, exhibited a broad XPS peak at approximately 169.6 eV, suggesting the presence of S with a relatively high valence number. This S peak with a higher BE may be attributed to naturally oxidized S,[2,3] which corresponds to S with a higher valence number. This is consistent with the Mo 3d doublet with a higher BE than the pristine MoS_2 peaks, as discussed in Figure 2e in the main text, suggesting the formation of Mo oxide. Importantly, the XPS spectra of the MoS_2 SE sample after limited oxidation under ambient conditions (sample B) exhibit S 2p core levels without additional features, indicating the absence of detectable interfacial chemical reactions.

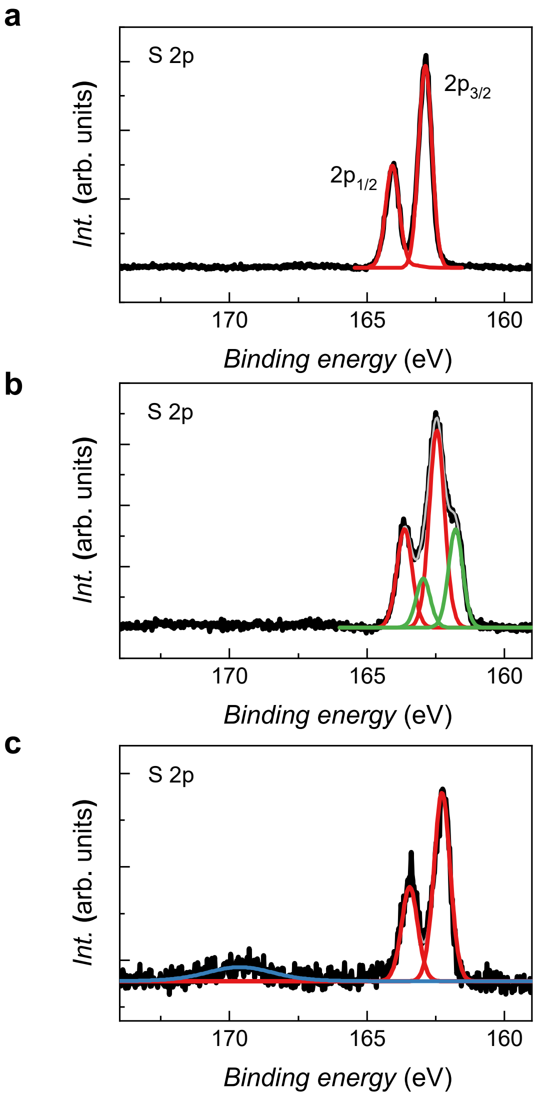

Figure S2. S 2p spectra of the higher binding energy (BE) regions of (a) sample A, (b) sample B, and (c) sample C in the main text. Sample C exhibited a broad XPS peak at approximately 169 eV after prolonged oxidation in ambient condition for 20 min, suggesting the presence of S with a relatively high valence number.

S3. The XPS analysis of the controlled MoS_2 SE sample with Cr/Au capping layer

To further support our claim that the negligible amount of oxidized states exists at the MoS_2 SE, we show a controlled MoS_2 SE sample etched by same method with Cr/Au capping layer instead of pure Au. Figure S3a and S3b show the Mo 3d and S 2p spectra of the control Cr-MoS_2 SE sample with periodic structure, respectively, revealing dominant Mo 3d and S 2p doublets. The Mo $3d_{5/2}$ doublet at 229.3 eV (red) can be attributed predominantly to bulk MoS_2. Similar to sample D in the main text, the well-resolved doublet (green) with a BE ≈ 0.7 eV lower than that of the doublet corresponding to the underlying bulk MoS_2 in both Mo 3d and S 2p spectra is attributed to the substoichiometric MoS_x layer. Importantly, the Mo XPS peak show absence of the MoO_x peak. The XPS analysis of the controlled MoS_2 SE sample with Cr/Au capping layer further supports our findings that MoS_2 SE exhibits negligible oxidized states in which the SE is etched by SF_6.

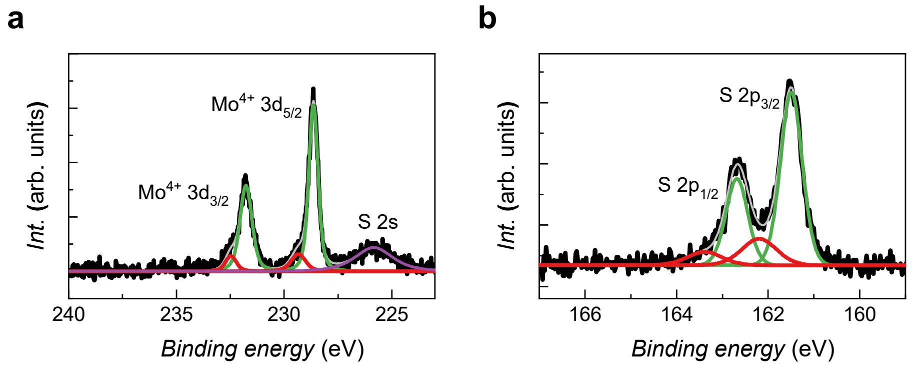

Figure S3. (a) Mo 3d and (b) S 2p spectra for a control MoS_2 SE sample with Cr/Au capping layer.

S4. Fabrication of the SE-contacted MoS_2 transistors

To fabricate SE-contacted MoS_2 transistors, we first produced a heterostructure consisting of stacked h-BN/MoS_2/h-BN layers. The bottom h-BN flakes were mechanically exfoliated onto poly(dimethylsiloxane) (PDMS) on glass slides and subsequently transferred to SiO_2 (285 nm)/Si substrates. After the samples were immersed in acetone and isopropyl alcohol (IPA), they were annealed in a furnace to remove the PDMS residue.[4] Both the MoS_2 and top h-BN flakes were subsequently mechanically exfoliated onto a PDMS and transferred onto the bottom h-BN flakes to complete the h-BN/MoS_2/h-BN heterostructures. The MoS_2 surfaces were cleaned by immersing the samples in acetone and IPA after MoS_2 was stacked onto the bottom h-BN layer. We evaluated the quality of the MoS_2 flakes via photoluminescence spectroscopy to confirm that crystalline degradation did not occur during the cleaning process.

We employed e-beam lithography to define the pattern for the SE contact on the MoS_2 transistors. The staked h-BN/MoS_2/h-BN samples were placed on a triangular quartz base at a 45° angle, which is enclosed within a Faraday cage to control the plasma etching direction. A mixture of SF_6 and Ar (1:10) was used in the plasma etching process with a base pressure of approximately 60 hPa. The metallic Au electrodes (60 nm) were subsequently deposited via e-beam evaporation under a base pressure of 1×10^{-8} Torr. The Au contact was selected for its low chemical reactivity for reproducible SE contact properties. For the left and right pairs of electrodes shown in Figure 3d in the main text, we conducted separate deposition procedures for each pair of contacts to fabricate SE contacts facing the center of the device.

Figure 4a, 4b, 4c in the main text are discussed for relatively high carrier density at $V_{GS} = 60$ V. Figure S4 shows the transfer curve of the SE-contacted ML MoS_2 transistor (sample D) at $T = 290$ K up to 60 V. The data is fundamentally the same as Figure 3f in the main text.

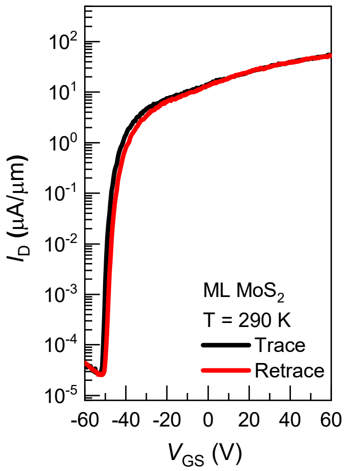

Figure S4. (a) Transfer characteristics for the ML MoS_2 SE-contacted device.

S5. Slanted edge of h-BN characterized by SEM

To ensure the SE structure, we characterized the h-BN test samples etched by SF_6/Ar plasma in a Faraday cage by SEM. As shown in the SEM image in Figure S5, a clear SE structure can be seen adjacent to the unetched h-BN flake to the left (gray region). On the right side, all of the h-BN layers were completely etched, leaving the SiO_2/Si substrate unetched with scattered h-BN residue. To fabricate the ML MoS_2 transistors encapsulated by h-BN, the SE etching conditions were mainly determined by h-BN owing to the dominant thicknesses of the top and bottom h-BN layers.

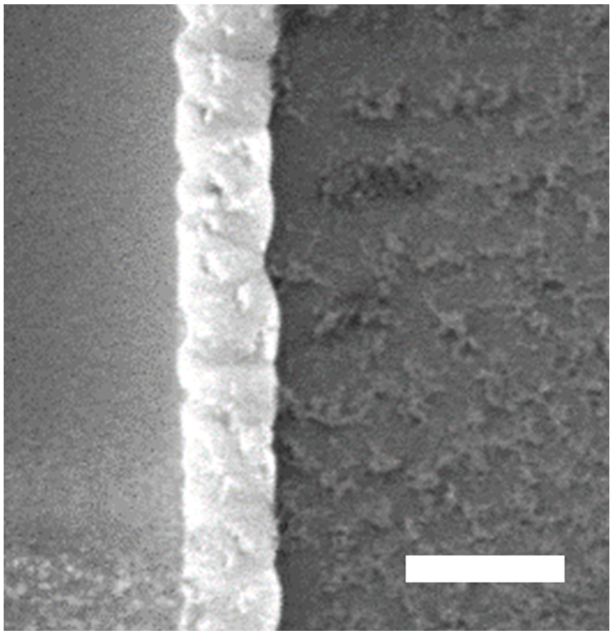

Figure S5. SEM image of the h-BN edge processed by directionally angled etching. The slanted edge is shown in the middle region with brighter contrast. The left and right sides of the slanted edge are the bulk h-BN and SiO_2 surfaces, respectively. The scale bar is 2 μm.

S6 Hybrid current/thermal annealing of the SE-contacted MoS_2 transistors

After fabrication of the SE-contacted MoS_2 transistors, a current/thermal annealing process was employed to significantly increase the drain current. Figure S6 shows the progressing transfer curve of a typical SE-contacted MoS_2 transistor at $T = 450$ K. Before current/thermal annealing, the device only slightly turns on within a range of $V_{GS} - V_{th} < 30$ V. With repeated application of high V_{GS}, the on-current is greatly enhanced, which is accompanied by suppression of the current fluctuations. Neither an individual current nor thermal annealing can alter device performance, making hybrid current/thermal annealing an effective measure to improve contact. Notably, the hysteresis is small even without annealing, indicating a clean interface upon completion of device fabrication.

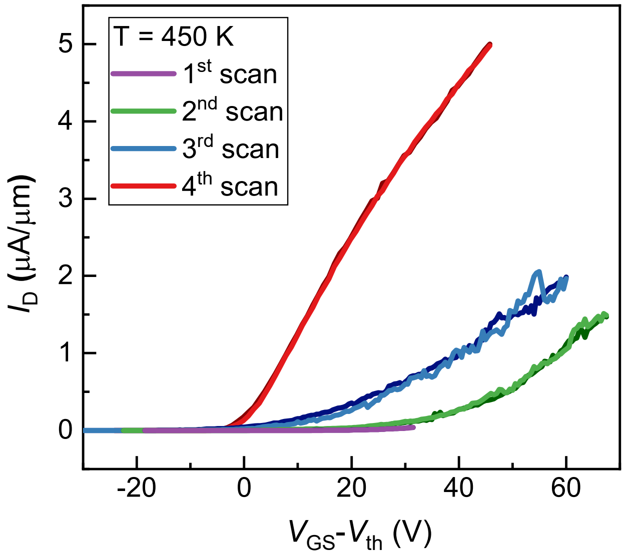

Figure S6. Transfer curve of a typical SE-contacted MoS_2 transistor at $T = 450$ K. The channel current increases with consecutive current/thermal annealing cycles.

To gain further insight into the Au–MoS_2 SE contact upon hybrid current/thermal annealing, the interfacial chemistry is analyzed through XPS spectra of Au-covered MoS_2 SE structure. It is noted that only thermal effect is considered due to the limitation of the MoS_2 SE samples for XPS measurement. Figure S7a and S7b presents the Mo 3d and S 2p spectra, respectively, of the Au–MoS_2 SE samples subjected to vacuum annealing. After metal deposition and lift-off process, the Au–MoS_2 SE sample was annealed under vacuum at 1×10^{-8} Torr at 200 °C for 6 hours. These spectra were compared with those in Figure 2c and 2d in the main text for the Au–MoS_2 SE samples without vacuum annealing. As shown in Figure S7a, the Mo $3d_{5/2}$ doublets at 229.8 eV and 229.0 eV were attributed to bulk MoS_2 and the substoichiometric MoS_x layer, respectively. These assignments are similar to those for sample B in the main text. The ratio of the core level peak areas corresponding to Mo $3d_{5/2}$ doublet of MoS_x layer to that of bulk MoS_2, $A(MoS_x)/A(MoS_2)$, for the Au–MoS_2 SE samples with vacuum annealing was 0.27, whereas it was 0.4 for the Au–MoS_2 SE samples without vacuum annealing, as shown in Figure S7c. This indicates decreased thickness of the substoichiometric MoS_x layer. The difference in BE between the two doublets was 0.8 eV, yielding a stoichiometry of approximately 1.34 on the basis of the estimation described in the main text. Compared with the stoichiometry of 1.5 for the Au–MoS_2 SE samples without vacuum annealing, as shown in Figure S7d, the stoichiometry decreases with vacuum annealing. The change of XPS spectra before and after thermal annealing indicates that the substoichiometric MoS_x layer becomes thinner with higher density of vacancy, as depicted in the schematic in Figure S7e. This change of structure after vacuum annealing may be attributed to that the sulfur atoms within the less chemically stable substoichiometric MoS_x layer migrate toward the bulk MoS_2, leading to its reconstruction but increasing the number of sulfur vacancies on the surface. Importantly, the thinning of the substoichiometric MoS_x layer is in line with the reduction in the contact barrier and increase in current upon hybrid current/thermal annealing of the SE-contacted MoS_2 transistors.

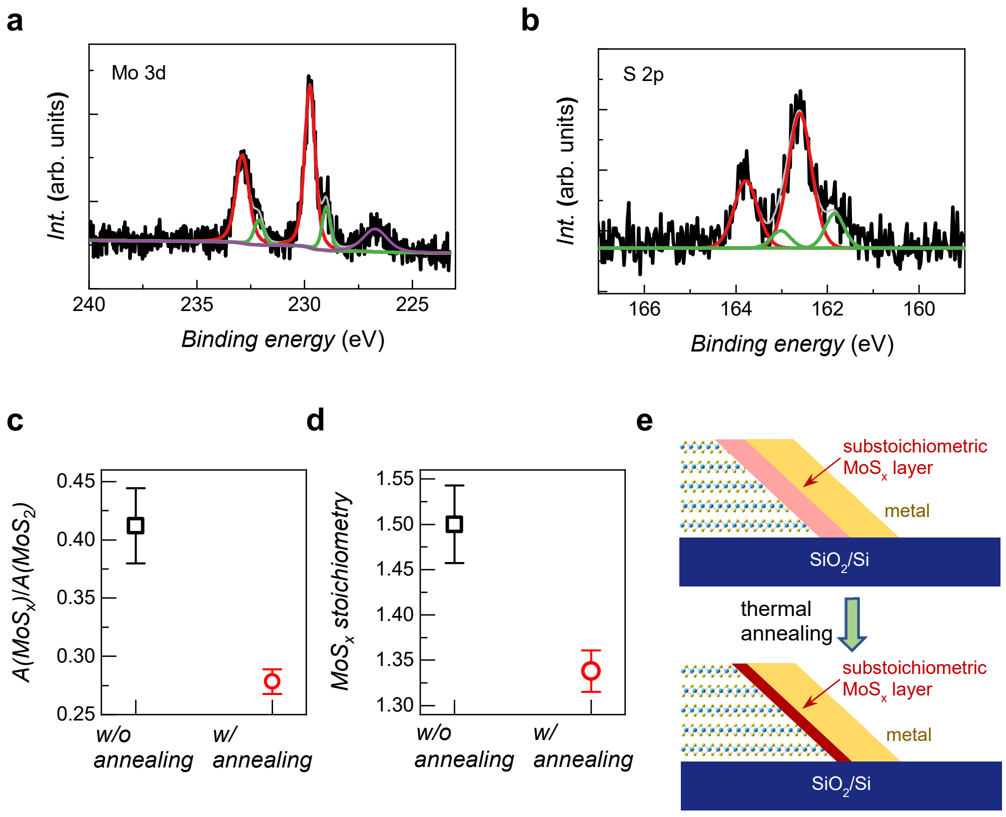

Figure S7. (a) Mo 3d and (b) S 2p XPS spectra of the Au–MoS_2 SE samples after vacuum annealing. (c) Area ratio $A(MoS_x)/A(MoS_2)$ before and after vacuum annealing. (d) MoS_x stoichiometry before and after vacuum annealing. (e) Schematics of substoichiometric MoS_x layer before and after vacuum annealing.

S7. Electrical transport mechanism

To extract the Schottky barrier height (Φ_B), we employ the thermionic emission model[5] to analyze the T-dependent transfer curve, where the drain current injected through a reverse-biased SB can be written as

$$I_D = A^* T^\alpha \exp\left[-\frac{E_A}{k_B T}\right]\left[1 - \exp(-\frac{qV_{DS}}{k_B T})\right] \quad (1)$$

where A^* is the Richardson constant, k_B is the Boltzmann constant, and α is an exponent (3/2 for 2D semiconductors). Notably, the T-dependent transfer curve is well described by the thermionic emission model in the temperature range of 200 to 260 K. Figure S8a shows the logarithmic transfer curve for SE-contacted ML MoS_2 (sample D) at different temperatures, where I_D increases with increasing temperature. Figure S8b shows the Arrhenius plot $I_D/T^{3/2}$ as a function of $1000/T$ corresponding to the on state of sample D with a V_G between -20 and 10 V. The transport characteristics do not follow thermionic behavior, as evidenced by the positive slope over the temperature range of 200 to 320 K. This suggests that the electron transport across the contact is governed by the tunneling mechanism and is not affected by the Schottky barrier, as depicted in Figure 4e in the main text.

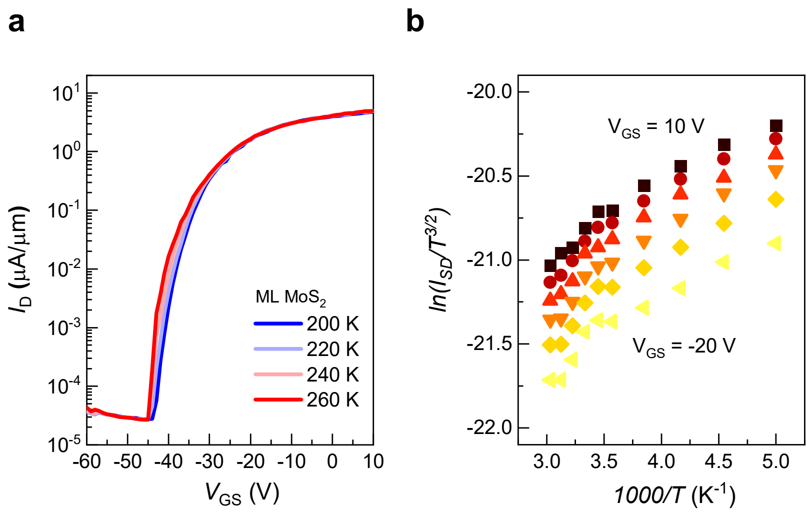

Figure S8. (a) Temperature dependence of the logarithmic transfer curves of an SE-contacted ML MoS_2 transistor (sample D in the main text) at $V_{DS} = 4$ V. (b) Arrhenius plot $I_D/T^{3/2}$ as a function of $1000/T$ corresponding to the on state of sample D with V_G between -20 and 10 V. The transport characteristics do not follow thermionic behavior, as evidenced by the positive slope over the temperature range of 200 to 320 K.

S8. Using Simmon's model for tunneling current

To further elaborate on the effect of channel length on the transport behavior, we note that the output characteristics reflect the contact dominated transport at high carrier density. In the main text, we have shown the non-linear output characteristics (Figure 4c) consistent with the tunneling behavior. Here, we further analyze the output characteristics by Simmon's model for tunneling current [6], and the fitting result is shown in Figure S9. It is found that the output characteristics can be well described by the tunneling model, further substantiating our interpretation. Therefore, by using long channel MoS_2 devices, the contact transport behavior can be revealed by varying V_{DS} while lowering the contribution from channel resistance to total resistance at high carrier density.

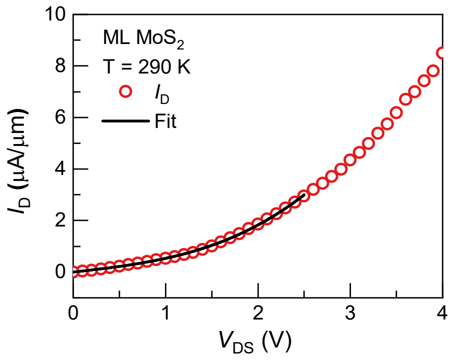

Figure S9. (a) Fitting by tunneling model for output curve of the ML MoS_2 SE device.

S9. Power dependence of the PL on the ML MoS_2 adjacent to the SE contact of the ML MoS_2 transistor

The photoluminescence spectra were acquired with a CW diode laser at 532 nm under a vacuum of 1×10^{-5} Torr at 300 K. Figure S10a and Figure S10b show the excitation power dependence of the PL peaks measured at d = 0 and d = 3 μm, respectively, which is defined as the distance from the SE contact, as described in the main text. All the power dependences of the intensities of the A^-, A^0 and B peaks are linear, confirming the excitonic behavior of these exciton species. On the basis of the power dependence of the PL, the scenario in which the PL originates from defects, which are exhibited as sublinear behavior,[7] is excluded, suggesting a clean MoS_2 SE contact interface.

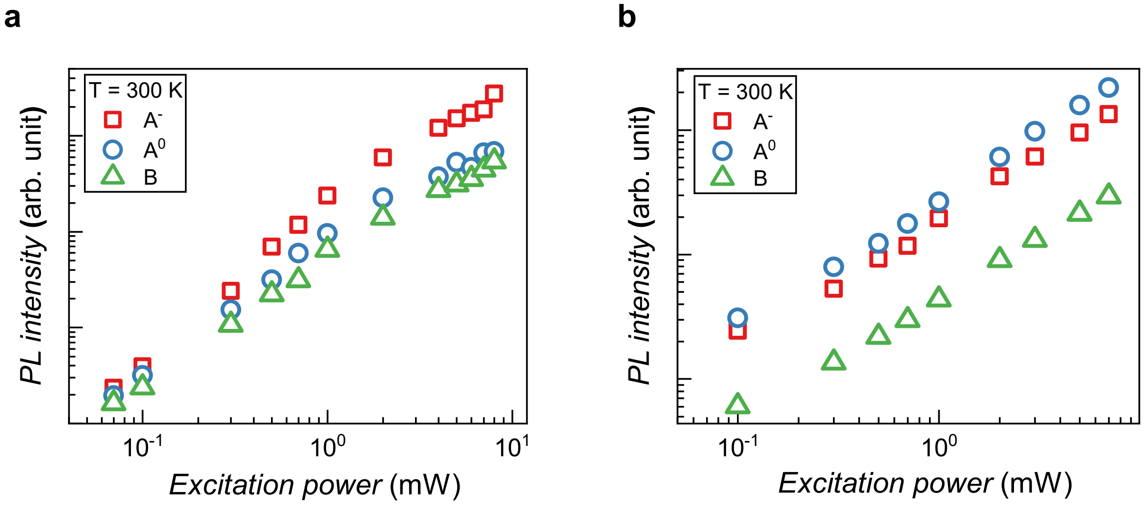

Figure S10. Power dependence of the PL on the SE-contacted ML MoS_2 device. The PL was measured (a) at the SE contact (d = 0 μm) and (b) away from the SE contact (d = 3 μm), as depicted in the main text. The linear behavior in the low-excitation-power region confirms the excitonic behavior of these exciton species.

References

1 Bengtsson, Lars, *Electrical Measurement Techniques: For the Physics Laboratory.* (Spinger, 2024).

2 Escalera-López, D. *et al.* Hydrogen evolution enhancement of ultra-low loading, size-selected molybdenum sulfide nanoclusters by sulfur enrichment. *Appl Catal B-Environ* **235**, 84-91 (2018).

3 Lin, Q. *et al.* Molybdenum disulfide with enlarged interlayer spacing decorated on reduced graphene oxide for efficient electrocatalytic hydrogen evolution. *J Mater Sci* **55**, 6637-6647 (2020).

4 Jain, A. *et al.* Minimizing residues and strain in 2D materials transferred from PDMS. *Nanotechnology* **29**, 265203 (2018).

5 Allain, A., Kang, J. H., Banerjee, K. & Kis, A. Electrical contacts to two-dimensional semiconductors. *Nat Mater* **14**, 1195-1205 (2015).

6 Simmons, J. G. Generalized Formula for Electric Tunnel Effect between Similar Electrodes Separated by a Thin Insulating Film. *J Appl Phys* **34**, 1793-& (1963).

7 Chakraborty, C., Goodfellow, K. M. & Vamivakas, A. N. Localized emission from defects in $MoSe_2$ layers. *Opt Mater Express* **6**, 2081-2087 (2016).
